# A Home-Based Intervention to Improve Adherence to the 24-Hour Movement Guidelines in Young Children: Protocol for a Mobile App–Based Randomized Control Trial

**DOI:** 10.2196/75621

**Published:** 2026-01-29

**Authors:** Chelsea L Kracht, Jerica M Berge, Monique LeBlanc, Robert L Newton Jr, Ryan E Rhodes, Grace Bolamperti, Madigan Snodgrass, Leanne M Redman

**Affiliations:** 1 Department of Internal Medicine University of Kansas Medical Center Kansas City, KS United States; 2 Department of Family Medicine University of Colorado Anschutz Medical Campus Aurora, CO United States; 3 Department of Psychology Southeastern Louisiana University Hammond, LA United States; 4 Population and Public Health Division Pennington Biomedical Research Center Baton Rouge, LA United States; 5 School of Exercise Science, Physical and Health Education University of Victoria Victoria, BC Canada; 6 Clinical Sciences Division Pennington Biomedical Research Center Baton Rouge, LA United States

**Keywords:** screen time, sitting time, movement behavior, sleep, physical activity, adherence

## Abstract

**Background:**

One in 10 preschoolers (aged 3-4 y) meet the three 24-hour Movement Guidelines, that is, (1) physical activity, (2) sedentary screen time, and (3) sleep.

**Objective:**

The overarching aim of this study is to evaluate the effectiveness and feasibility of a 12-week mobile health home-based intervention on 24-hour movement behaviors in preschoolers who meet few guidelines (zero or 1 guideline).

**Methods:**

We will conduct a 12-week randomized controlled trial with a wait-list control in 80 families (40 per arm). Preliminary studies in this population informed intervention app content, features, and app development. Behavior change theories, including transfer theory and the multi-process action control framework, helped inform content presentation and topics. Primary outcomes include device-based and parent-report measures of 24-hour movement behaviors, and the secondary outcome is the feasibility and acceptability of the app. Exploratory outcomes include preschoolers’ cognitive and motor skills, changes within the home environment, and behavioral control processes.

**Results:**

This 2-phase study (K99/R00) received initial funding in March 2022, and preliminary studies were concluded in December 2023. The main grant received institutional review board approval in April 2024, and the grant funding began in May 2024. The study was registered in Clinical Trials in October 2024 and enrolled its first participant in January 2025. As of October 2025, the study has enrolled 39 families. We anticipate the trial will be completed in late 2026.

**Conclusions:**

This research is designed to test a novel approach to improve all three 24-hour movement behaviors in preschoolers in home settings by using a mobile app. Results from this study will have implications for future 24-hour movement interventions, our understanding of improving all 3 behaviors, and ultimately, improvements in preschoolers’ health.

**Trial Registration:**

Clinicaltrials.gov NCT06667661; https://clinicaltrials.gov/study/NCT06667661

**International Registered Report Identifier (IRRID):**

DERR1-10.2196/75621

## Introduction

In 2019, the World Health Organization created the 24-hour Movement Guidelines [1], which provide recommendations for preschoolers’ (aged 3-4 y) physical activity (PA; ≥3 h/d of total PA, including 1 hour of moderate-to-vigorous physical activity [MVPA]), sleep (10-13 h/d), and sedentary behavior (≤1 h/d of sedentary screen time) to promote physical and mental development [2-6]. Only 15% of preschoolers meet all 3 recommendations, and thus many preschoolers are at risk for compromised development and health [7,8]. For example, in a prospective cohort study, preschoolers who met zero or 1 guideline had lower motor skills 1 year later compared with those who met all 3 guidelines [5]. These alarming rates of poor adherence reveal an urgent need for effective interventions that support parents in promoting adherence to all 3 recommendations and thus prevent adverse health outcomes over the long term.

Current interventions overlook the importance of the home and family setting that influences when, how, and why all three 24-hour movement behaviors occur. To the authors’ knowledge, there is one 24-hour movement intervention that primarily focused on the parent, rather than the home environment, and demonstrated limited effectiveness [9]. The home environment has a significant contextual influence on child’s 24-hour movement behaviors, as illustrated by poor adherence rates to the 24-hour Movement Guidelines during the COVID-19 pandemic [10]. Facilitating changes to multiple movement behaviors within the home environment can rely on behavior change theory, and gaps between intention and behavior often prevent behavior change [11]. To change behaviors, attitudes, habits, and behavioral regulation patterns must be formed [12], including action plans (eg, planning and enlisting support) [13]. One framework that supports motivational (eg, attitude and perceptions of control), self-regulatory (eg, action plans), and habit theory is the multi-process action control (M-PAC) framework. The M-PAC framework combines aspects of behavior change together to form and translate intentions into long-term behavior change [14,15]. The M-PAC model can be applied to PA approaches. For example, family planning for PA can result in changes to family PA [16]. Bridging the intention-to-behavior gap in child movement behaviors also provides an unprecedented opportunity to leverage the transfer theory of behavior change, whereby changes in one behavior lead to changes in another behavior [17,18]. Habit formation, M-PAC, and transfer theory can be used in a 24-hour movement behavior intervention to change multiple behaviors in young children for long-term adherence to all 3 recommendations.

A mobile health (mHealth) modality (eg, mobile apps) can address behavioral patterns by delivering context-based information to parents. Parent-based mobile apps have been effective in reducing preschooler sugar-sweetened beverage consumption [19] but have yet to be translated to improving 24-hour movement behaviors. This paucity is demonstrated in a recent systematic review of digital health interventions for preschool PA, where the authors found 8 interventions in total, and only 1 was a parent-based mobile health app [20]. This mobile health app demonstrated effectiveness on child health behaviors, including PA, suggesting these modalities can be used to reach parents and impact child health behaviors [21]. Together, these modalities provide an opportunity to build upon previous work and change movement behaviors, especially for those at risk for poor adherence to the 24-hour Movement Guidelines.

The overarching aim is to conduct a study to evaluate the effectiveness and feasibility of a 12-week mHealth home-based intervention on 24-hour movement behaviors in preschoolers who meet few guidelines (zero or 1 guideline). Primary outcomes include device-based and self-report measures of 24-hour movement behaviors, and the secondary outcome is the feasibility and acceptability of the app. Exploratory outcomes include preschoolers’ cognitive and motor skills, changes within the home environment, and behavioral control processes. We hypothesize that children will increase the number of recommendations obtained, with 35% of preschoolers meeting 24-hour Movement Guidelines (all 3 recommendations), based on other routine-focused interventions [22,23]. We also hypothesize parents will report the intervention is feasible (≥4.0/5.0 on Likert scale).

## Methods

### Study Design

This study, *Shining Star*, is a randomized controlled trial with a waitlist control design. This protocol follows the SPIRIT (Standard Protocol Items: Recommendations for Interventional Trials) reporting guidelines for randomized trials (Multimedia Appendix 1).

### Participants, Eligibility, and Recruitment

To be included, parents must report that their preschooler meets zero or 1 guideline for PA, sleep, and screen time as defined by the World Health Organization at the screening call [1]. Zero or 1 guideline was chosen as existing prospective studies demonstrate meeting 1 or zero guidelines is associated with lower motor skills and cognitive development compared with meeting all 3 guidelines [5,24]. Based on work conducted before the COVID-19 pandemic, we anticipated 15%-20% of preschoolers would qualify [5], with similar proportions found in a preschooler-focused systematic review [4]. Adherence to the 24-hour Movement Guidelines by parent report was chosen to reduce the burden, align with a survey study that informed analysis [25], and ensure the inclusion of the target population. The PA guideline is defined as ≥180 min/d of total PA, including ≥60 min/d of MVPA. The sleep guideline is defined as 10-13 h/d. The sedentary behavior guideline is defined ≤1 h/d of sedentary screen time.

Parents must also agree to use their mobile phone for the study duration (approximately 12 weeks), download the mobile app onto their phone, be fluent in English, and be willing to travel to the research site to complete study visits. Exclusion criteria are mobility limitations as reported by the parent, meeting 2 or 3 of the 24-hour movement guidelines, sibling or household member participating in the study, and parents reporting the preschooler will not wear a device on their wrist.

This study seeks 80 families, with 40 families per arm. Parents of preschoolers (aged 3-4 y) are recruited from a metropolitan area of a Midwest US state. We plan to recruit through the institution’s research-based list-serves (including local children’s hospitals), a local advertisement through social media, blogs, magazines, community-based efforts (eg, childcare centers and local events), and at local medical centers. Recruitment efforts include flyers and paid advertisements.

### Preliminary Studies

In total, 2 separate studies were conducted to inform this intervention and mobile app. First, there was a cross-sectional study of 42 parents of preschoolers (24/42, 57% non-Hispanic White and 21/42, 51% male) who met few guidelines (zero or one of the 24-hour Movement Guidelines) who completed semistructured interviews; this information was used to inform mobile app content and describe existing resources and interest in a mobile app. For this cross-sectional study, we obtained written informed consent. The second study included 4 focus groups of 18 parents of preschoolers (10/18, 55% non-Hispanic White and 7/18, 38% male) who also met a few guidelines. We obtained verbal consent before participation for the second study. These focus groups aimed to solicit feedback on the core components and initial design of a 24-hour movement behavior intervention delivered through a mobile app.

In semistructured interviews, parents were asked about barriers and facilitators to their preschooler’s and family’s 24-hour movement behaviors, existing use of mobile apps and other resources, and interest in mobile app features to promote any of the preschoolers’ 24-hour movement behaviors. Interview transcripts were reviewed to identify common barriers and facilitators for each behavior within the home environment, and app interest. These barriers and facilitators were aligned with existing multilevel frameworks [26,27], which included levels at the child, family, home environment, and macroenvironment. For study content, we specifically focused on factors that may inform a 12-week home-based intervention, rather than policy or system-level changes.

**Figure 1:**
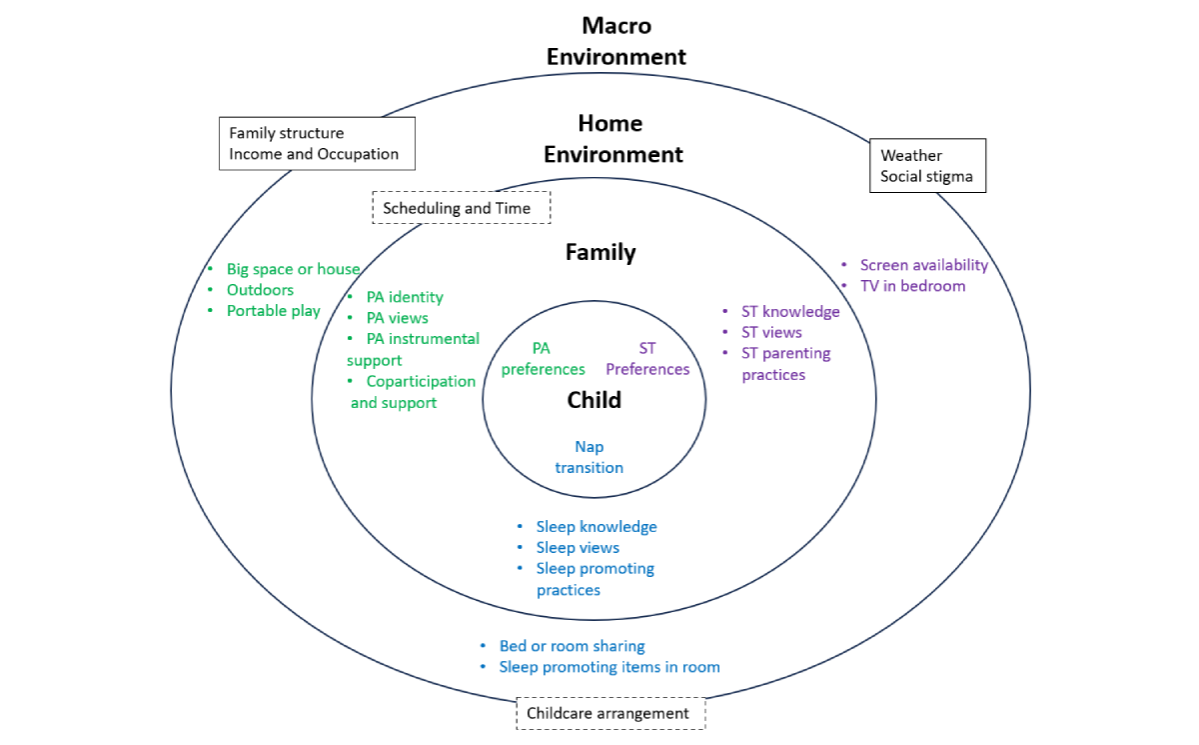
Conceptual model of nonmodifiable and modifiable factors that influence child’s 24-hour movement behaviors within the home (each ring represents a level of influence). PA: physical activity; ST: screen time. Solid boxes represent nonmodifiable factors. Hashed boxed represent modifiable factors that influence all behaviors.

The nonmodifiable and modifiable factors are displayed in Figure 1 and described in detail in Table S1 in Multimedia Appendix 2. Nonmodifiable factors of family structure, income or occupation, weather, social stigma, and childcare arrangement were macroenvironment factors that impacted all 3 levels. A modifiable factor that impacted both the family and home environment, and all movement behaviors was scheduling and time management. In general, the remaining single behavior modifiable factors were interrelated with other behavior factors, especially between screen time and sleep. For example, parents may report that allowing screen time (screen time parenting practice) is part of the bedtime routine and helps their child wind down (sleep-promoting practice). These nonmodifiable and modifiable items then informed content matter and behavioral targets for the intervention. For example, in the week 9 lesson, Indoor Activities for MVPA, the content matter discusses the benefits of being outdoors (modifiable: outdoors) and preparing and planning for being active in less-than-desirable weather (nonmodifiable: weather).

### App Development

#### Study 1: Semistructured Interviews

Most parents (25/42, 59%) reported that they did not use apps to support any of the child’s 24-hour movement behaviors, but did use apps to manage a home or family schedule (27/42, 64%), namely, a calendar app or shared to-do list. Parents reported many sources of information on each 24-hour movement behavior, which included the internet, family, and friends (Table S2 in Multimedia Appendix 2). Some reported using no resources for any of their preschooler’s 24-hour movement behaviors (10/42, 23%). The majority (36/42, 85%) expressed interest in receiving information on their preschooler’s PA, sleep, and screen time from a mobile app. Few (5/42, 12%) were unsure about their interest in a mobile app as they did not want more device notifications. Only 1 participant expressed that this app would not change their behavior.

#### Study 2: Focus Groups

Among 2 focus groups (n=10), parents were interested in the rationale for changing behaviors, opportunities to track their child’s behaviors, gamification, and relating to other parents during the program. Importantly, parents were interested in information for changing child’s PA and screen time to improve child’s sleep, rather than focusing on all 3 behaviors at one time. Parents were interested in having opportunities to use preplanned and self-selected action plans to facilitate change. Some parents mentioned they would like a child-facing component for the app; this feature was ultimately not included in the final mobile app as it may indirectly impact preschooler’s sedentary screen time.

In our third and fourth focus groups (n=8), we presented the created mobile app “*Shining Star*” to families for their feedback on design, features, and intervention components. The app was designed using the Pathverse mobile app, a no-code platform for researchers to create mobile health apps to facilitate behavior change [28]. Families liked the length and design of lessons, various options to set action plans, internal gamification, and the anonymous chat forum to connect with other parents. Overall, there was excitement for the mHealth app.

### Intervention

#### Content

The 12-week intervention follows a multibehavior change process or transfer theory, whereby changing one behavior may lead to changes in another behavior [17,18]. This multibehavior framework was derived from semistructured interviews and was supported by focus group results, where parents were interested in learning about 1 behavior at a time, rather than all 3 at once. The first few lessons focus mainly on sleep and screen time activities based on the following: (1) focus group participants were interested in learning more about sleep, (2) many semistructured interview participants reported screen time during the bedtime routine, and (3) most participants would likely not meet the screen time guidelines and so may benefit from these early changes. After the focus on sleep and screen time during the first 6-8 weeks, the final weeks are focused on positive PA changes with the framing of making “daytime” changes to improve sleep. Given that PA is one of the more difficult behaviors to change, but parents recognize its impact on sleep, this behavior was chosen to be presented last.

As illustrated in Table 1, content was created based on behavior change techniques [29] and mechanism of action (MoA) [30,31] that would facilitate habit formation and align with the M-PAC framework. Accordingly, parents were provided with content that addressed reflective, regulatory, and reflexive processes. To address each part of M-PAC, aligning BCTs demonstrating links to appropriate MoA was identified through an online tool (The Theory and Techniques Tool). Using week 1 as an example, BCTs of “BCIO007063: information on health consequences” and “BCIO007068: salience of health consequences” were chosen for the MoAs of knowledge, attitude toward health behavior, and perceived susceptibility; these BCTs and MoA align with the M-PAC construct “instrumental attitude.” Later in week 3, “BCIO007080: prompts and cues” then aligns with the MoA of behavioral cuing and the M-PAC construct of habit. Each week, parents have the option to set goals and action plans supporting the M-PAC construct of behavioral regulation. Parents also have the option to receive points for completing the lesson and meeting a goal, which aligns with BCIO0007257: provide positive material consequence for behavior, and the MoA of reinforcement.

**Table 1:** Shining Star intervention content and techniques to facilitate behavior change.

Phase, week, focus, behavior, and major topic		BCT^a^ label and ID [29]	Mechanism of action [30,31]	Multiprocess action control construct	Conceptual model items addressed
**Night**					
	Week 1: all behaviors—24-h movement to improve sleep	007063: Information on health consequences 007068: Salience of consequences 007024: Self-monitoring of behavior	Knowledge Attitude toward health behavior Perceived susceptibility Behavioral regulation	Instrumental attitude Behavioral regulation	ST^b^ knowledge PA^c^ views Sleep knowledge Social stigma Sleep promoting items in room TV in bedroom Screen availability Sleep parenting practices Portable play items Outdoors Big space or house
	Week 2: sleep—bedtime routines (who, what, and when)	007065: Inform about emotional consequences BCT 007058: Instruction on how to perform the behavior 007055: Demonstration of the behavior	Knowledge Skill Capabilities	Affective judgment Perceived capability	Sleep knowledge Sleep promoting practices ST knowledge ST parenting practices
	Week 3: sleep—how to make the new bedtime routine, routine	007096: Context specific repetition of behavior 007094: Practice behavior 007080: Prompt intended action	Behavioral cuing (problem solving)	Habit formation Behavioral regulation	Scheduling time, chaos Sleep promoting practices
	Week 4: sleep or screen time—bedroom environment	050348 Restructuring the physical environment 050331: Remove averse stimulus 007156: Add objects to the environment 007080 Prompts and cues	Environmental context and resources Behavioral cuing	Perceived opportunity Affective judgment Habit	Television in bedroom Sleep promoting items in room Bed or room sharing Screen availability
	Week 5: sleep— parent sleep habits	007063: Information on health consequences 007068: Salience of consequences 007057: Draw attention to incompatible beliefs 007159 Affirm valued self-identity	Knowledge Attitude toward health behavior Perceived susceptibility	Instrumental attitude Identity	Sleep knowledge Sleep views
	Week 6: sleep—how family members can support goals	050346: Directly restructure the social environment BCT 007030: Advise to seek instrumental support 0007257: Provide positive material consequence for behavior	Social context and resources Behavioral cuing Social influences Reinforcement	Perceived opportunity Identity Behavioral regulation Affective judgment	Sleep promoting practices Bed or room sharing ST parenting practices Screen availability Childcare arrangement Family structure
	Week 7: sleep—reassessment of goals	007063: Information on health consequences 007068: Salience of consequences 007058: Instruction on how to perform the behavior 007024: Self-monitoring of behavior	Knowledge Attitude toward health behavior Perceived susceptibility Skill Capabilities Behavioral regulation	Instrumental attitude Perceived capability Behavioral regulation Perceived opportunity	ST knowledge PA views Sleep knowledge Social stigma Sleep promoting practices ST parenting practices Sleep promoting items in room Television in bedroom Screen availability Sleep parenting practices Portable play items Outdoors
**Day**					
	Week 8: screen time—screen-limiting during the day	007063: Information on health consequences 007068: Salience of consequences 007058: Instruct on how to perform behavior 007055: Demonstrate the behavior	Knowledge Attitude toward health behavior Perceived susceptibility Skill Belief about capabilities	Instrumental attitude Perceived capability	ST knowledge ST views ST parenting practices Screen availability Television in bedroom Portable play items Outdoors Big space or house
	Week 9: PA—indoor activities for MVPA^d^	007058: Instruct on how to perform behavior 007055: Demonstrate the behavior 050348 Restructuring the physical environment	Skill Belief about capabilities Environmental context and resources Behavioral cuing	Perceived capability Behavioral regulation Perceived opportunity	Weather Big space or house PA instrumental support Coparticipation ST parenting practices PA or ST preferences Portable play items Outdoors Television in bedroom
	Week 10: PA— being an active family	007064: inform about social consequences 007057: Draw attention to incompatible beliefs 007159 Affirm valued self-identity	Knowledge Belief about capabilities Attitude toward behavior	Instrumental attitude Identity	PA instrumental support Coparticipation PA or ST preferences PA identity
	Week 11: PA—setting up for success or finding “time” for PA	007064: inform about social consequences 007096: Context specific repetition of behavior 007094: Practice Behavior 007080: Prompt intended action 007058: Instruct on how to perform behavior	Knowledge Belief about capabilities Behavioral cuing Behavioral regulation	Instrumental attitude Identity Habit formation	PA instrumental support Coparticipation PA identity PA or ST preferences Scheduling time Childcare arrangement
	Week 12: PA—overall review	007063: Information on health consequences 007068: Salience of consequences 007058: Instruction on how to perform the behavior 007024: Self-monitoring of behavior 0007257: Provide positive material consequence for behavior	Knowledge Attitude toward health behavior Perceived susceptibility Skill Capabilities Behavioral regulation Reinforcement	Instrumental attitude Perceived capability Behavioral regulation Affective judgment	ST knowledge PA views Sleep knowledge Social stigma Sleep promoting practices ST parenting practices PA identity PA instrumental support Coparticipation Sleep promoting items in room Television in bedroom Screen availability Portable play items Outdoors Big space or house
**All weeks**					
	Weeks 1-12	007003: Set behavior goal	Intention	Behavioral regulation	Various
	Weeks 1-12	007008: Goal strategize BCT	Beliefs about capabilities Behavioral regulation	Behavioral regulation	Various
	Weeks 1-12	007010: Action planning	Behavioral cuing	Behavioral regulation	Various
	Weeks 1-12	0007257: Provide positive material consequence for behavior	Reinforcement	Affective judgment	Various

^a^BCT: behavior change technique.

^b^ST: screen time.

^c^PA: physical activity.

^d^MVPA: moderate-to-vigorous physical activity.

#### App Features

**Figure 2:**
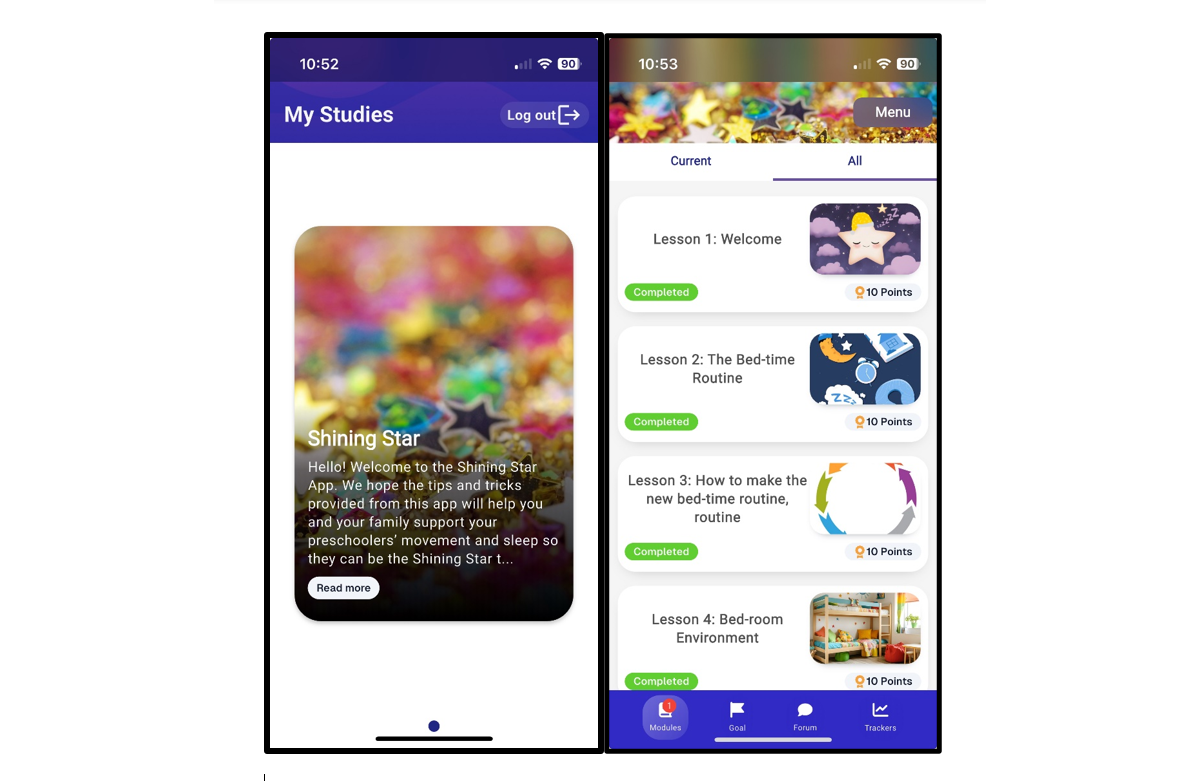
Screenshots of the Shining Star study welcome page and table of contents.

Using the Pathverse platform [28], we created weekly lessons and behavior-related goals. Each lesson is concise for readability (<500 characters) with a quiz, a suggested weekly goal, and links to additional resources. Features within the app to facilitate behavior change included gamification of completing lessons and weekly goals, an optional tracker for child behaviors (eg, sleep and going outside), an optional anonymous chat form to relate to other parents with preset chat topics (eg, local events), and a resources tab with additional research-based information on the importance of 24-hour movement behaviors. A screenshot of the app and the table of contents are presented in Figure 2.

The Pathverse platform is a secure and Health Insurance Portability and Accountability Act (HIPAA)–compliant platform with end-to-end encryption to deliver health content, monitor health behaviors, and support behavior change. To protect child and family information, the platform uses a confidential generic email address supplied by the study team. Content posted within the app is monitored regularly, with concerning or irrelevant posts addressed in a timely manner. No concerning posts have been detected by October 2025. The Pathverse app (which houses the intervention and its content) is available for download on the App Store and is compatible with both iOS (Apple Inc) and Android (Google), and can be downloaded on many digital devices (eg, cellphones and tablets). For this study, all parents are asked to download it onto their personal cellphones, and no parents, as of October 2025, have opted to download the app onto another device.

### Procedures

**Figure 3:**
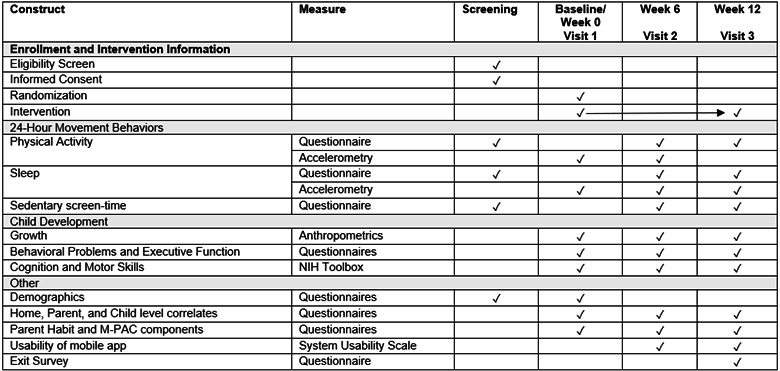
Schedule of enrollment, intervention, and assessments during study. Feasibility and acceptability will be assessed weekly through the mobile app, and control participants are given access to the intervention after completing all week 12 measures, including 14-day accelerometry. M-PAC: multiple process action control; NIH: National Institutes of Health.

An overview of enrollment, intervention, and assessments is provided in Figure 3. Harms and adverse events will be assessed at each visit. Study assessments are low-risk; thus, we anticipate few study-related harms. As this study is ongoing, assessments are described in the present tense.

#### Screening

Interested parents complete a web screening tool using a secure online platform [32], which includes information on the preschooler’s age and sex, language fluency, and interest and ability to download the app. Potential participants who met screening criteria are contacted for a phone call, during which a trained staff member inquires about the ability to travel to visits, the preschooler’s ability to wear a device on their wrist, and the preschooler’s habitual PA, sedentary screen time, and sleep. Eligible families are then scheduled for their baseline visit (week 0).

#### Baseline Visit (Week 0)

At this visit (approximately 60-90 min), the study is described in a child-friendly manner to the preschooler, and the parent provides written informed consent. After consent is obtained, preschooler’s height and weight are measured. The preschooler completes developmental tests with a trained researcher, and the parent completes questionnaires. Finally, an accelerometer is secured to the preschooler’s wrist to wear for 2 weeks. The parent is instructed to return the accelerometer, an activity or sleep log, and a home inventory of equipment related to 24-hour movement behaviors after 2 weeks. At the end of the visit, the family is randomized.

If randomized to the intervention, the *Shining Star* mobile app is downloaded onto the parent’s phone. A trained staff member provides a tutorial to explain the mobile app use and set mobile app reminders. The parent’s first weekly lesson and app features are available that day.

#### Follow-up Visits (Week 6 and Week 12)

At the second visit (approximately 60-90 min; week 6), families return to complete preschooler anthropometrics, preschooler development tests, and parent questionnaires. Procedures are completed similarly to the baseline visit. Preschoolers are fitted with an accelerometer, and parents are asked to return the device, activity or sleep log, and home inventory after 2 weeks of wear. The trained staff answer any questions on the mobile app for families in the intervention group.

At the final visit (approximately 60-90 min; week 12), families return to complete preschooler anthropometrics, preschooler development tests, and parent questionnaires. Procedures are completed similarly to the baseline visit. Preschoolers are fitted with an accelerometer, and parents are asked to return the device, activity or sleep log, and home inventory after 2 weeks of wear. Parents in the intervention are allowed to delete the mobile app or continue viewing the content. Parents randomized to the control are allowed to download the mobile app, receive a tutorial, and access all mobile app lessons and features.

### Measures

#### Primary Outcome

Our primary outcome is adherence to all 3 of the 24-hour Movement Guidelines. Preschoolers’ PA and sleep were assessed in 15-second epochs using the Actigraph GT3X+ accelerometer. Preschoolers wear this device on their wrist using a Velcro band, only removing it for water-based activities (eg, bath). Accelerometer data will be evaluated using the Sadeh algorithm to detect sleep periods and compared with the parent-report of sleep [33]. Periods of ≥20 minutes of continuous zeroes during the day will be removed as nonwear time [34,35]. Validated cut points for wrist measurement in this age range will be used for sedentary behavior (0-328 counts per 5 seconds), light PA (329-1392 counts per 5 seconds), and MVPA (≥1393 counts per 5 seconds) [36]. Total PA is the sum of light PA and MVPA. Preschoolers with ≥3 days (at least 1 weekday and 1 weekend day) of ≥10 hours of wear, and 3 days of sleep, will be included for analysis, similar to another wrist-worn protocol [37], and is the estimated minimum for accelerometer wear in this age range [38,39]. To capture adherence to the sedentary screen time guideline, parents are asked, “On an average 24-hour period, how much time does your child spend using electronic screen devices (such as a smartphone, tablet, video game, or watch television or movies, videos on the internet) while they are sitting or lying down?” with a free response option. This question is conducted at screening and subsequent visits and was chosen to align with surveillance efforts of movement behaviors [40]. This questionnaire reflects the previous week before accelerometer wear. The sum of guidelines obtained based on these measures, and if the participant meets all 3 guidelines, will be used for analysis.

#### Secondary Outcome

Feasibility and acceptability are assessed by a weekly survey within the app. The parent reports how difficult or easy it was to achieve the goal, such as removing TV from the bedroom (Likert scale responses: range 1-5). Parents also rate their satisfaction with the app with regard to design, appeal, and functionality, and complete the System Usability Scale at week 6 and week 12, which can be used to assess overall usability of the app [41]. At the final visit, participants randomized to the intervention complete a 15-item survey to assess the acceptability and usability of app features and content, and overall participation. Responses from these questions will be combined with app usage data to evaluate the performance of these components for future iterations.

#### Exploratory Outcomes

##### Parent Report of 24-Hour Guideline Adherence

Parents will report how many days per week (range: 0-7) their child obtains at least 180 min/d of total PA and at least 60 min/d of MVPA. Staff members describe total PA as including any activity, such as slow or easy movement, based on direct report observations of preschooler’s movement [42]. These may include walking, light dancing, playing catch, and easy bike riding. MVPA includes any “huff and puff” activity that increases their heart rate, such as playing a sport, more intense dancing, and running. If parents have trouble discerning days, the staff will ask them about the past week. Furthermore, parents will be asked on average how much overnight sleep and time their child spends napping, in separate questions with free response options, which are summed for a total amount of sleep during the day. The sleep questions have demonstrated moderate correlations with accelerometry measures [43]. Parents are also asked about screen time (refer to “Primary Outcomes” section). We chose these questions based on national surveys of child PA [44] and surveillance efforts of child movement behaviors [40,45]. This information is gathered during the phone screening process, where trained staff members ensure parents consider both weekdays and weekends, and any changes due to nontypical weeks, to obtain accurate habitual behavior amounts. Screening questions related to total PA, MVPA, and sleep are repeated at week 6 and week 12.

##### Anthropometrics

The child’s height (cm) and weight (kg) are objectively measured using standard operating procedures with a mounted stadiometer and portable scale. BMI (weight/height^2^) will be calculated and compared with national growth standards to calculate BMI *z* score and categorize weight status [46].

##### Cognitive and Motor Skills

These constructs are measured using the recent update of the National Institutes of Health Toolbox V3 [47]. Preschoolers complete the cognitive tasks validated for this age range and consolidated from the previous version (V2) [48,49]. The tests and associated constructs are picture vocabulary test (language), visual reasoning (nonverbal and visual reasoning), picture sequence memory test (episodic memory), and speed matching (processing speed) [50]. The preschooler also completes the National Institutes of Health Toolbox Early Childhood Motor Battery, which includes the tests and constructs as follows: 9-hole pegboard dexterity test (fine motor), grip strength (muscular strength), standing balance (balance), and 2-minute walk endurance test (muscular endurance). The preschooler completed the cognitive measures on a tablet, while the motor skills were completed with a trained administrator.

##### Behavioral Problems and Executive Function

Preschoolers’ executive function and behavioral problems are assessed during this study. Parents complete questions with Likert scale responses from the Behavioral Assessment System for Children (3rd edition) related to child behavioral problems, and the Behavioral Rating Inventory of Executive Function Preschool for executive function [51], which will then be used to calculate the preschool child’s T-score for analysis. Both questionnaires are valid tools and have shown to be useful tools for assessing child development [51,52].

##### Descriptive Characteristics

Home, parent, and child-level correlates, along with parent habit and M-PAC components, are collected**.** Home space and equipment are assessed by a parent report inventory of preschooler-appropriate availability and accessibility of PA (eg, jump rope) and media (eg, television) equipment, room design, and sleep components. This updated inventory is based on validated inventories in parents of older children [53] and preschoolers [54,55]. Household organization was assessed using the Confusion, Hubbub, and Order Scale, which is a 15-item measure assessing disturbance, organization, and noise levels in the home. It has been validated in parents of young children [56].

Parent-level correlates include parent support for PA, parent screen time practices, and bedtime and family routines. Parents complete a 4-item questionnaire to assess parental support of child PA based on a validated questionnaire in this age range [57], which includes core support elements, including encouragement, logistics, and coparticipation [58]. Parents report their perceived influence on their child’s screen media use and limit setting of screen media from a validated questionnaire for parents of preschool children (12 questions total). These scales had high internal consistency (0.84-0.86) and correlation with child BMI and screen time [59]. Parents are asked 7 questions on family regularity, including having a bedtime ritual and regularity of family meals over the last week, based on a validated questionnaire for preschoolers [60].

Child-level correlates include solely child preferences. Parents complete questions, including “*when outside, my child prefers*” with a list of activity options, “*my child’s physical activity is limited due to their interest or motivation*,” and their child’s preference for PA in their free time, with Likert scale options for each question. These scales had high internal consistency (0.60) and correlation with child screen time [59].

Finally, parental support for PA and sleep and limiting screen time is examined using self-report items of habit formation to establish routines for each behavior [61], and an adapted questionnaire measuring intention and behavioral support for all 3 movement behaviors based on the reflective, regulatory, and reflexive parts of the M-PAC model [11].

### Stipends and Incentives

Intervention participants can redeem points achieved through engagement with the mobile app for up to US $75 worth of incentives. Examples of incentives are activities to do at home to encourage PA (eg, child wearable device) and sleep (eg, sound machine), and reduce screen time (eg, puzzles) [11].

### Analytical Approach

#### Randomization and Blinding

Participants are randomized to intervention or control in a 1:1 ratio with block randomization stratified by age and sex, with a random allocation process developed by a biostatistician. Due to the nature of the study, staff administering the first visit are unblinded to group assignment.

##### Aim 1: Meeting 24-Hour Movement Guidelines

We will compare the proportion of intervention and control group participants meeting all 3 recommendations at 12 weeks using a chi-square analysis.

##### Aim 2: Feasibility

We will average the Likert scale responses (ranges 1.0-5.0) across all weeks completed (up to 12). Feasibility and acceptability will be achieved if the average response is ≥4.0/5.0 for each category.

#### Sample Size and Power Calculation

Our analysis is based on a previous home-based routine-based multibehavior intervention in preschool children, which sought to improve similar behaviors, and reported an increase in the proportion of children meeting the screen time guideline increased from 5% to 30% over 12 weeks [23]. Based on our past studies, we estimate that few will meet the screen time guideline [25,62], like the beginning metric of the routine-based study. Given the overlap in content between the routine-based study and this study, we hypothesize that we can also improve the screen time guideline and potentially the remaining behaviors within the same time frame. A minimally important difference in 24-hour movement behaviors is not clear as of October 2025, as there are no other 24-hour movement guideline adherence studies. A sample size of 30 families per arm will provide 80% power to detect a meaningful difference in the proportion of control (1/30, 5%, no change from baseline) and intervention group (10/30, 35%) participants meeting all 3 recommendations at the 12-week visit. Assuming a dropout rate of 25%, we will recruit 40 families per arm (80 total). This power and difference can also be achieved with a minimum of 24 participants per group; while 30 participants per group will have 63.5% power to detect a difference between 5% and 25%, meeting all 3 recommendations; and a 99% power to detect a difference between 5% and 20%, meeting all 3 recommendations. Statistical significance will be set at *P*=.05 for all aim-related analyses.

#### Exploratory Aims

We will also conduct an intent-to-treat analysis using a log-binomial regression or Poisson with robust variance to adjust for baseline recommendation number and randomization strata (age and sex). Linear regression will also be used to compare changes in exploratory outcomes between treatment and control groups with adjustment for baseline values. If demographic differences, such as income, are found between the 2 groups, we will conduct a sensitivity analysis with these variables for adjustment. These exploratory outcomes include, in priority order: number of recommendations obtained (range: 0-3), individual 24-hour movement behaviors, anthropometry, early child motor battery scores, early child cognitive battery scores, executive functioning scores, and behavioral problem scores. Descriptive characteristics will also be explored, such as PA and accessibility score, household chaos score, parental support of PA, parental perceived influence on screen time, family regularity index, child preferences for PA, and theoretical component changes (eg, intention and behavioral support for PA). We will also explore the ideal change in behaviors using isotemporal substitution analysis, whereby evaluating substitution of one behavior (eg, sedentary behavior) for another (eg, MVPA) with change in child developmental outcomes.

Furthermore, we will examine the relationship between meeting specific recommendations and child outcomes, with a mediator of theoretical component changes (eg, habit formation scores). Mediation analysis will be conducted using the PROCESS vs 3.5 macro with 10,000 bootstrap intervals with unstandardized estimates and CIs [63]. These exploratory outcome testing will be subject to the Benjamini-Hochberg adjustment for multiple comparisons [64].

As for missing data, we will describe differences in baseline characteristics between participants with complete versus incomplete follow-up. Sensitivity analyses based on multiple imputation methods will be conducted to examine the robustness of our findings with respect to missing data.

#### Recruitment Plans

We will use various existing modalities of recruitment in the selected area to ensure the recruitment amount is achieved. As part of this 12-week study, participants are contacted every 2 weeks or up to 1 month to schedule visits and pick up accelerometers. All available data will be sought in the case of discontinuation after visit 1, with the priority of 24-hour movement behavior data (questionnaire and accelerometry), other questionnaires, then anthropometry, cognition and motor skill assessments.

#### Data Management Plan

All participants will be given an ID as part of the screening process. This ID will be stored in a secure database at the University of Kansas Medical Center. Only approved project staff will have access to the server. Data obtained during study visits and as part of the mobile app use will be reported as tabular data and stored in a secure electronic data capture system. All subject-level clinical data will be preserved and shared. Shared data will be deidentified, and the original data will be maintained at the investigator’s institution. The minimal risk of this study and its short-term nature precluded appointment of a data monitoring committee.

### Ethical Considerations

The University of Kansas Medical Center Institutional Review Board approved this study in April 2024 (approval 00160250). The Pennington Biomedical Research Center Institutional Review Board approved both the studies that were conducted for this protocol (approvals 2021-053 and 2023-011). The informed consent specified that every effort would be made to maintain participant confidentiality, such as deidentifying private information, and participants had the option to opt out of the study. Participants are paid up to US $200 for completing all study visits and receive small child toys (<US $5) at each complete visit.

## Results

This study recruitment began in January 2025 and is anticipated to conclude in December 2026. As of October 2025, 39 families have been enrolled and randomized into the study, with 19 families in the intervention arm and 20 in the wait-list control arm. As of October 2025, there has been one loss after randomization. Furthermore, 28 families have completed their final visit, and no adverse events have been reported. Descriptive and analytic results are expected to be published in the year 2027 and reported within the trial registry.

## Discussion

### Principal Findings

This study aims to improve adherence to all three 24-hour Movement Guidelines in preschoolers who meet few guidelines using a theoretically informed, home-based, mobile app. Our preliminary studies in the target population demonstrate multilevel considerations to improve all 3 behaviors within the home, which can be improved through existing frameworks of multibehavior change, habit formation, behavior adoption, and behavior maintenance. Using mHealth technology allows information to be provided to parents within the most applicable setting for 24-hour movement behaviors, the home environment. Together, a rigorously designed behavior change intervention with thorough preliminary work has resulted in the *Shining Star* study.

This study’s use of a mobile app presents challenges and opportunities to impact preschooler’s 24-hour movement behaviors. The majority of parents in our focus groups were interested in receiving information about their child’s 24-hour movement behaviors from a mobile app. The mobile app platform may reduce travel burden and reach parents in the home setting; the mobile app may also promote additional parental screen time. Given parental screen time is an established correlate of child screen time [59,65] and identified as a factor in this study’s conceptual model, we did not include the parent-suggested child-facing component in the app. Rather, the intervention content and features focus on creating habits, goals, and planning to improve each 24-hour movement behavior; these components have all been associated with reducing child screen time in a meta-analysis of 204 interventions [66]. Nonetheless, parental screen time practices are collected as part of this study to examine whether the current app impacted a key parent 24-hour movement behavior.

### Comparison With Previous Work

To the authors’ knowledge, there is only one other 24-hour movement-focused intervention, as the current 24-hour movement scientific landscape is predominantly observational studies [9]. That study was also a 12-week randomized controlled trial with wait list control in 147 young children, and it demonstrated significant decreases in screen time but no intervention effect in other behaviors [9,67]. Major differences between the past trial and this trial include (1) population (this trial: 3-4 y who meet 0 or 1 guideline, past trial: 3-5 y who meet 0-2 guidelines), (2) intervention approaches (multibehavior and transfer theory vs all 3 behaviors at once and integrated), (3) outcomes (adherence to 24-hour Movement Guidelines vs Activity Sleep Index), and (4) intervention methods (all mobile app vs internet-based education and online workshops) [9]. It is unclear if this study’s different populations, intervention approaches, modalities, and outcomes will result in significant results, but there will still be a noticeable contrast.

### Strengths and Limitations

The strengths of this study are a theoretically based intervention in an important population and outcome for improving child health outcomes. This study uses a mobile app, which also allows for measuring engagement and usage of the intervention. Limitations of the study pertain to measurement and generalizability. A potential limitation is the use of parent self-report of child 24-hour movement behaviors for inclusion, which may overreport or underreport certain behaviors, and a lack of overlap in screen time measure questions with accelerometer wear. This study addresses this limitation by using validated individual questions and having thoroughly trained staff conduct these assessments, so accurate measures can be obtained. Furthermore, this use of parent-reported guideline adherence for screening and accelerometer data for outcome measures may misclassify participants and potentially weaken effect estimates. Currently, there is no full 24-hour movement behavior self or parent-report measure for this age range, and there are a limited number of valid self-report tools for 24-hour movement behaviors in general [68]. Another limitation is the generalizability of this study. This intervention is based on preliminary data in the United States, and sleep habits and screen time may significantly differ across the globe. Thus, content and potential results may not translate to other populations [69]. Moreover, this study solely includes English-speaking individuals, as app content was not translated to other languages before study commencement.

### Implications

If successful, this study has multiple implications for future research and practice. First, these results may posit using similar transfer theory approaches for improving all 3 movement behaviors, rather than all at once as conducted in another 24-hour movement trial in preschoolers [9]. Second, these results may support the use of mHealth apps for improving children’s movement behaviors and adopting other evidence-based information to such platforms to reach parents. Finally, significant results support testing this intervention in a larger trial of diverse participants, as children from low-income and minority backgrounds are at greater risk for not meeting these recommendations, as they are more likely to have excess screen time [70], short sleep [71], and less PA relative to counterparts [72]. Findings from the larger trial and this study would then inform meaningful changes in preschoolers meeting 24-hour movement recommendations and translating this mobile app in pediatricians’ and sleep health professionals’ practices.

### Conclusions

In summary, this paper describes the creation of a novel approach to improve all three 24-hour movement behaviors in home settings using a mobile app. This study will use a unique approach to improve multiple levels of influence and ultimately preschooler behavior. Results from this study have implications for future 24-hour movement interventions, our understanding of improving all 3 behaviors, and ultimately, improvements in preschoolers’ health.

## Appendix

Multimedia Appendix 1 SPIRIT checklist.

Multimedia Appendix 2 Components on the 24-hour movement behaviors in the home environment conceptual model and resources to promote adequate 24-hour movement behaviors provided by parents.

## Abbreviations

mHealth: mobile health
HIPAA: Health Insurance Portability and Accountability Act
MoA: mechanism of action
M-PAC: multi-process action control
MVPA: moderate-to-vigorous physical activity
PA: physical activity
SPIRIT: Standard Protocol Items: Recommendations for Interventional Trials

## Data Availability

Preliminary study data analyzed for this paper is available upon reasonable request. Final data generated from this study is available from authors upon reasonable request and will be submitted to a National Institutes of Health–approved repository.
