# Supplementary material for: A Home-Based Intervention to Improve Adherence to the 24-Hour Movement Guidelines in Young Children: Protocol for a Mobile App–Based Randomized Control Trial
Source: JMIR Res Protoc. 2026 Jan 29;15:e75621. doi: 10.2196/75621 (PMC12902759; doi:10.2196/75621)
Supplement: Multimedia Appendix 2 [file resprot_v15i1e75621_app2.pdf]

| Table S1. Description of components on 24-hour movement behaviors in home environment conceptual model | |
|---|---|
| Construct | Description of impact on 24-hour movement behaviors |
| ***Macro Environment – non modifiable*** | |
| Family structure | The amount of people and relationship of people within the household impacted 24-hour movement behaviors. This included parents, partners, siblings, and local grandparents. Single parents specifically described challenges not having an additional support during daily tasks compared to two parent households. |
| Income/ Occupation/ Childcare arrangement | Lower income individuals described challenges enrolling children in organized sports, finding affordable physical activity options, and childcare compared to higher income families. Parent's described nontraditional or extended hours impacting the availability and context of activities they did with their children. For example, some parents were tired from difficult jobs or stayed up late finishing work and had less energy the next day to engage in supportive parenting practices. These changes are indirectly impacted the amount of physical activity, screen-time, and sleep for children across the day. |
| Weather | Rain, heat, and cold were all described as barriers to physical activity. Some parents did describe that their child participated in activity outside regardless of the rain. However, the heat was consistently mentioned as a facilitator of indoor activities and parents described "beating the heat" by engaging in sedentary screen-time. |
| Social Stigma | Parent's felt that their use of screen-time was constantly being judged as too little or too much. Parents described other people's, including friends and family, liberal or tight use of screen-time making it harder to enforce their own rules. In these situations, they felt pressured to use screen-time like others. |
| ***Home environment – physical*** | |
| Big space/ house | Having a large space within the house was described as a facilitator of physical activity, as children had room to run around inside or had an official playroom with toys. Parents with a small space had less options for indoor physical activity and had to utilize other spaces (outdoors or other person's house). Parents who had less space described the woes of higher night-time noise and proximity to others impacting children's sleep. |
| Outdoors | Parents described being outdoors as a main facilitator of physical activity. Families with big backyards described using this space for physical activity throughout the week, as children could play with others and their play items outdoors. The only hinderance to outdoor time was parents could not always readily view them while they were playing outdoors, or parent's own injury or preferences may preclude them from going outdoors. Proximity to parks and outdoor spaces was also mentioned as a facilitator to physical activity, as it allowed an additional outdoor option. |
| Portable play items | Items such as push mowers, bicycles, and other child driven moveable items were described as facilitators to either indoor or outdoor physical activity. These items were described as they could be used outside, or in tandem with other family members physical activity pursuits. Parents emphasized a variety and incorporating them into daily or weekly activities was helpful for child physical activity. |
| Screen availability | TVs in the bedroom and tablet access were described as facilitators to screen-time. TV in the bedroom lead to more screen-time at night, either when it was used as a sleep aid or the child turned it on sometime later at night. This practice increased screen-time then impacted total sleep time. Other screens within the home also impacted child screen-time since these screens were usually on from other family |

| | |
|---|---|
| | members use. As for tablets, parents described the child's tablet being available in differing situations at home, in the car, and other social gatherings (e.g., church, restaurant). Few parents described allowing their child to use their own phone. |
| Bed/room sharing | Bed and room sharing was described as both a barrier and facilitator to sleep. Bed sharing included the child leaving their bed in the middle of the night to go sleep with parents, for various or unknown reasons. Some parents tried to get the child to go back in their own room, which decreased the child's total sleep time. Room sharing was described in both positive and negative light, as having a room companion who was a "good" sleeper helped promote sleeping, but sometimes younger siblings waking up or trying to play with the preschooler impacted them going to bed. |
| Sleep promoting items in room | Noise machines, books, and blackout curtains were described as sleep promoting items within the room, which positively impacted child sleep. Some parents described a light and noise system (e.g., Hatch TM) that allowed children to know when to get up. |
| ***Family – modifiable factors that influence all behaviors*** | |
| Scheduling/time/ chaos | Parent, sibling, and child schedules not aligning were described as a major barrier to physical activity and sleep, and promoter of screen-time. Parent's mentioned using screen-time to occupy children while they engaged in household chores or tended to sibling's needs. Also, parent's non-traditional schedules and sibling after school activities led to more screen-time as the child spent extended time in the car, and family members needed screen-time to wind down from the later home arrival. This late arrival also delayed or compromised child bed-time routines and bedtime. Chaos was somewhat referenced during night-time activities when parents were trying to get everyone coordinated within the home to go to bed. |
| ***Family environment*** | |
| Physical Activity identity | A physical activity facilitator was parent's describing their family as an "active" or a "sports" family. Parent's took pride in their children participating in sports, sometimes from their own past sport participation. |
| Physical Activity views | Parent's who viewed physical activity as important for child development were more likely to facilitate child physical activity. Alternatively, parents who felt overwhelmed by their children's constant activity and needed a break were less likely to promote physical activity. Sometimes parent's confused "busy" or "occupied" for physical activity, such as engaging in a play kitchen or doing crafts, which are activities that do not elicit exercise-like activity. |
| Physical activity instrumental support | Parents taking children to sports, going to parks, and paying for active options were facilitators of child physical activity. Some of these paid options were organized sports or play gyms. |
| Co-participation/ support for physical activity | Parents, siblings, and dog participation were facilitators of child physical activity. This facilitation may be through directly engaging in activity or modelling physical activity. Parents usually described their partner as the one facilitating activity, rather than themselves due to their own injury or existing household demands. Commonly, siblings and dogs facilitated physical activity while outdoors. |
| Screen-time knowledge | Parent's own knowledge on screen-time, including the detrimental effects of screen-time on child development, were barriers to screen-time. No parents described knowing the guidelines for screen-time in this age range but described an ideal amount to be around 1-1.5 hours a day. Others with less knowledge on the impacts of screen-time used adult guidelines of 2-3 hours per day for their child. |
| Screen-time views | Parent's screen-time views were a major contributor to child screen-time throughout the day. Some parents who felt that children needed to use screens to get ready for school and adjust to the digital world early. Some parents felt children were learning quite a lot from screen-time, but described small achievements that may have also |

| | |
|---|---|
| | been obtained in childcare. Parent's definition of what counted as screen-time differed, as some did not count TVs being on in the background or co-participation in sibling's screen-time. Parents all had various views of what was considered educational screen-time across devices. |
| Screen-time parenting practices | Limiting screen-time, either through setting timers on devices or forbidding it during certain times of day, were major barriers to screen-time. Not setting limits, having very high limits (e.g., 3 hours per day), or only forbidding double device usage did not deter child screen-time. Some parents viewed that their child didn't engage in as much screen-time as others, so were less motivated to engage in screen-time parenting practices. |
| Sleep knowledge | Parent's own knowledge of sleep, and its importance on child development was a major facilitator of child sleep. Parent's knowledge on sleep was from personal experiences with child, pediatricians, or other resources (e.g., articles or apps). These parents usually prioritized naps and other sleep promoting routines across the week and weekend. |
| Sleep views | Parent's own views sometimes were barriers to good sleep as some parents felt their child did not need a nap or that they were a good sleeper, so no additional changes were needed. Parents also described that if both parents prioritized sleep, it was easier to enforce a bedtime. |
| Sleep promoting practices | Parent's use of sleep promoting practices, such as a bed-time routine, reading before bedtime, a strict bedtime, and making a calm environment were helpful for children to fall asleep. Some parents mentioned using melatonin to aid in their child falling asleep. Some parents also used the TV as a night light, a noise machine, or to help children wind down in their bed. |
| ***Child*** | |
| Physical activity preferences | Parents described their child's preference for physical activity as a facilitator. Some children were described as naturally active, while others were described as preferring sedentary pursuits. Parents often compared their child's preferences to siblings or other children their age. |
| Screen-time preferences | Parents described their child's attachment to screen-time as a barrier to reducing screen-time. Some children preferred screen-time to physical activity. Parent's also described behavioral challenges when removing screen-time and asking children to engage in physical activity. |
| Nap transition | Parents described that children's own nap impacts their night-time sleep, in both positive and negative lights. Some parents noted that if they don't nap, they may be overtired later, having trouble going to bed, and wake up early. Others noted that if they nap at school, they stay up late, and is another hinderance to their sleep. |

| **Table S2. Resources to promote adequate 24-hour movement behaviors provided by parents (n=42)** | | |
|---|---|---|
| Response: Count | Options: Count | Overall Description |
| Yes: 29<br>(Many parents listed more than one resource) | Internet: 15<br>Family and Friends: 8<br>Community: 6<br>Existing Knowledge: 5<br>Pediatrician: 4<br>Other resources: 25 (Vroom vroom, mychart, books, hatch, youtube, pokemon go, newsletters, Outschool, sleep and activity trackers) | Parents' preferred 24-hour movement resource is the internet, specifically search engines such as Google because of the free and easy access to information. Some parents seeking information from other parents, however, many mentioned having different parenting styles than friends and family and having to take everything with a "grain of salt". Other resources varied significantly, from apps to promote movement (Pokemon Go), their own use of sleep and activity trackers, and some even mentioned books. |
| No: 10 | | Parents reported not previously using any resources to help their child be more active, sleep better, or watch less screens. Parents either did not explain their reason or claimed their child has never needed help with 24 hour movement. |
| Not asked by interviewer: 3 | | |
